# Company matters: The presence of other genotypes alters traits and intraspecific selection in an Arctic diatom under climate change

**DOI:** 10.1111/gcb.14675

**Published:** 2019-07-02

**Authors:** Klara K. E. Wolf, Elisa Romanelli, Björn Rost, Uwe John, Sinead Collins, Hannah Weigand, Clara J. M. Hoppe

**Affiliations:** ^1^ Marine Biogeosciences Alfred Wegener Institut – Helmholtz‐Zentrum für Polar‐ und Meeresforschung Bremerhaven Germany; ^2^ Marine Science Institute University of California Santa Barbara California; ^3^ University of Bremen Bremen Germany; ^4^ Helmholtz Institute for Functional Marine Biodiversity (HIFMB) Oldenburg Germany; ^5^ Institute of Evolutionary Biology, School of Biological Sciences University of Edinburgh Edinburgh UK; ^6^ Aquatic Ecosystem Research, Faculty of Biology University of Duisburg‐Essen Essen Germany

**Keywords:** allele‐specific qPCR, artificial population, genotypic interactions, intraspecific diversity, multiple stressors, ocean acidification, phenotypic plasticity, selection dynamics, strain sorting, warming

## Abstract

Arctic phytoplankton and their response to future conditions shape one of the most rapidly changing ecosystems on the planet. We tested how much the phenotypic responses of strains from the same Arctic diatom population diverge and whether the physiology and intraspecific composition of multistrain populations differs from expectations based on single strain traits. To this end, we conducted incubation experiments with the diatom *Thalassiosira hyalina* under present‐day and future temperature and pCO_2_ treatments. Six fresh isolates from the same Svalbard population were incubated as mono‐ and multistrain cultures. For the first time, we were able to closely follow intraspecific selection within an artificial population using microsatellites and allele‐specific quantitative PCR. Our results showed not only that there is substantial variation in how strains of the same species cope with the tested environments but also that changes in genotype composition, production rates, and cellular quotas in the multistrain cultures are not predictable from monoculture performance. Nevertheless, the physiological responses as well as strain composition of the artificial populations were highly reproducible within each environment. Interestingly, we only detected significant strain sorting in those populations exposed to the future treatment. This study illustrates that the genetic composition of populations can change on very short timescales through selection from the intraspecific standing stock, indicating the potential for rapid population level adaptation to climate change. We further show that individuals adjust their phenotype not only in response to their physicochemical but also to their biological surroundings. Such intraspecific interactions need to be understood in order to realistically predict ecosystem responses to global change.

## Graphical abstract

Six freshly isolated strains of the Arctic diatom *Thalassiosira hyalina* were incubated as mono‐ and multistrain cultures under different temperature and CO_2_ conditions. Although strains originated from the same water sample, monocultures showed large physiological diversity. When tested all together in multistrain cultures, selection dynamics as well as bulk physiology within these artificial populations differed fundamentally between the two treatments and diverged strongly from predictions based on monoculture traits. This suggests that cells change their phenotype depending on their biological surroundings and that such intraspecific interactions need to be better understood to predict future phytoplankton ecology from experimental data.

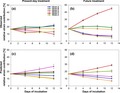

## 1 INTRODUCTION

Marine phytoplankton are not only the base of the oceanic food web but also the main driver of the biological carbon pump, which strongly influences the biogeochemical cycles in the oceans (Geider et al., 2001). Diatoms play a central role in these processes as they are the most important primary producers in the present‐day oceans and contribute disproportionally to the vertical carbon flux, especially during highly productive bloom events (Sarthou, Timmermans, Blain, & Tréguer, 2005). Therefore, their responses to rising temperatures and exponentially increasing CO_2_ concentrations are of great relevance for ecosystems as well as for climate feedbacks. The Arctic environment, which is changing far more rapidly than the global average (Miller et al., 2010), can provide a prime example of the ability or failure of organisms to respond to rapid environmental change.

Our attempts to understand and predict future phytoplankton community productivity and species composition often rely on the upscaling of single strain responses to environmental drivers as measured in laboratory experiments (e.g., Dutkiewicz et al., 2015). Such laboratory setups, however, have yielded varying results (Gao & Campbell, 2014), especially when compared with observations from studies using more complex assemblages (Sommer, Paul, & Moustaka‐Gouni, 2015; Tatters et al., 2018). Awareness of genotypic as well as phenotypic diversity within phytoplankton species has grown considerably in recent years (Alpermann, Tillmann, Beszteri, Cembella, & John, 2010; Brandenburg et al., 2018; Godhe & Rynearson, 2017; Hattich et al., 2017; Kremp et al., 2012; Pančić, Hansen, Tammilehto, & Lundholm, 2015; Wolf, Hoppe, & Rost, 2018) and may partly explain differences in results. With the recognition that trait diversity can be considerable within species, we now have to understand how knowledge gained in single strain studies can be applied in an ecological context that assumes or models multistrain communities (Follows & Dutkiewicz, 2011; Fontana, Thomas, Moldoveanu, Spaak, & Pomati, 2017; Kiørboe, Visser, & Andersen, 2018).

Understanding the relationships between responses of cultures containing a single genotype (hereafter referred to as monocultures) and populations made up of multiple genotypes is an important step toward predicting the responses of species, and eventually of entire communities, because effects of a rapidly changing environment may be amplified or buffered on any of these ecological levels. Thus far, knowledge about such interactions in phytoplankton mainly stems from research on different species in artificial assemblages, which are typically composed of very few long‐term established laboratory strains as representatives of each selected species. When questions are focused on understanding how species within a community may interact by using such setups, monoculture responses seem to predict the community outcomes fairly well (Low‐Décarie, Fussmann, & Bell, 2011; Pardew, Pimentel, & Low‐Decarie, 2018). However, from early agricultural research, we know, that a mix of species can have a different, often even higher yield than the best performing species grown in monoculture (“transgressive overyielding”; Trenbath, 1974). It has also been argued that biodiversity can have a buffering effect on both species persistence and community productivity called the “insurance effect” (e.g., Loreau, Mouquet, & Gonzalez, 2003; Yachi & Loreau, 1999). The result that population‐level responses (such as yield) can differ from the predicted outcome based on monoculture traits can be explained by a species' persistence being not only determined by the physicochemical conditions (i.e., the fundamental niche), but being also influenced by biological interactions (i.e., the realized niche, Elton, 1927), such as competition or facilitation (Bruno, Stachowicz, & Bertness, 2003; John et al., 2015). Biodiversity effects are often partitioned into “selection effects,” which apply if the community traits are driven by the dominance of a certain species, and “complementary effects,” which describe the (often positive) influence of species interactions (Cardinale et al., 2006; Loreau & Hector, 2001).

While the effects of interspecific diversity are reasonably well studied, the extent to which such concepts also apply to intraspecific diversity is only beginning to be discussed (e.g., Aguirre & Marshall, 2012; Reusch, Ehlers, Hämmerli, & Worm, 2005; Roger, Godhe, & Gamfeldt, 2012). Intraspecific (genotypic) diversity has been shown to affect the responses of phytoplankton populations in different ways. Some studies find that a diverse population performs as the mean of all strains in isolation (Hattich et al., 2017), while others indicate that they perform like the best performing component of the mix (Bell, 1991), which is then usually the dominant one. It has also been observed that a mixture of strains of the same species performs even better than the best one of its components in monoculture (John et al., 2015; Sjöqvist & Kremp, 2016; Vanelslander et al., 2009), which suggests that intraspecific interactions may influence strain traits. In other cases, mixtures of strains were found to underperform relative to monocultures (Collins, 2010). These contrasting results suggest that general mechanisms of intraspecific interactions are still poorly understood. Characterizing these interactions is limited methodologically as it is difficult to resolve the intraspecific genotype composition of microbial populations; they are typically inferred from subsamples of reisolated strains present at the end of an experiment, which allow one to draw conclusions regarding the endpoint of strain sorting, but does not resolve its temporal dynamics.

In this study, we focus on this knowledge gap by following intraspecific strain composition of a multistrain assemblage in different environments. Our objective was to characterize and compare the responses of different isolates of an Arctic diatom not only as single‐genotype monocultures but also when combined in an artificial multistrain population, whose genotypic composition and properties could be measured. The experimental setup described here was preceded by a natural community incubation of an Arctic phytoplankton assemblage. Aiming at resolving genotypes that may show the broad response range present within this population, we isolated several individual cells of our model species *Thalassiosira hyalina* from the final time‐point of two different treatments (i.e., selection environments) of the community incubation. We characterized six of these freshly established strains as monocultures under three environmental treatments of temperature and pCO_2_ conditions (“present‐day,” “warming,” and “future”) to investigate the extent of their plasticity as well as intraspecific variation in responses to climate change. From former experiments with this species (Wolf et al., 2018), we expected responses often found in diatoms: increased growth and productivity under higher temperature and variable, strain‐specific effects in the interaction with elevated pCO_2_. Subsequently, we combined these six strains into artificial multistrain assemblages and used microsatellite markers to measure the relative strain frequencies in the assemblages over time. This enabled us not only to evaluate the predictability of population productivity and bulk trait values of multistrain assemblages from monoculture traits but also to compare the selection dynamics that actually occurred in the multistrain assemblage with the predictions of population composition based on measurements made in monocultures.

## 2 MATERIALS AND METHODS

### 2.1 Strain origin and isolation

The six monocultures of *T. hyalina* investigated here were isolated from the final time‐point of an experiment with a natural Arctic phytoplankton spring community from the Kongsfjord, in Svalbard (mid‐fjord station KB3, 78°55′N, 11°56′E). The species was chosen due to its frequent dominance in Svalbard spring blooms (von Quillfeldt, 2000). The community incubation was conducted in April 2016 by applying combined pCO_2_ and temperature treatments under controlled light and nutrient conditions in a laboratory. The details of this experiment can be found in Hoppe, Wolf, Schuback, Tortell, and Rost (2018), where the experiment is referred to as KFb.

After 16–22 days of community incubation (duration depended on nutrient drawdown of the cultures), single cells of the diatom *T. hyalina* were isolated manually under a light microscope and washed three times in sterile seawater. Strains CPa24, CPa49, and CPb44 (hereafter referred to as strains A, B, and C) were isolated from bottles grown under the “present‐day” conditions at 1.8°C and ~320 µatm pCO_2_ (see Hoppe, Wolf, et al., 2018 for details). Strains WFa43, WFb25, and WFb51 (hereafter referred to as strains X, Y, and Z) were isolated from bottles under “future” conditions at 6.8°C and ~1,080 µatm pCO_2_. Single‐cell isolation was repeated after 10–14 days of growth in 48‐well plates at 6.8°C in 1–3 ml sterile nutrient‐enriched seawater. Each of the resulting monocultures was checked microscopically for contamination with other algal species and via microsatellites for other genotypes. The resulting stock cultures were maintained at 3°C and 5–10 μmol photons m^‐2^ s^‐1^ for about 9 months before the start of the experiment.

### 2.2 Experimental conditions

The six strains were incubated as monocultures in spring 2017 in 1 L glass bottles in semicontinuous dilute batch cultures (150–10,000 cells/ml, diluted every 2–5 days depending on cell density). Each strain was tested in a collapsed design matrix of three environmental treatments: at low temperature and pCO_2_ (2°C, 400 µatm) called “present‐day”; at high temperature and low pCO_2_ (7°C, 400 µatm) called “warming”; and at both high temperature and high pCO_2_ (7°C, 1,200 µatm) called “future.” Prior to the experimental phase, cultures were acclimated to treatment conditions for at least 6 days (>7 generations). The acclimation phase was considered to be completed when the mean of daily specific growth rates (µ, per day) during at least two consecutive dilution cycles of one replicate culture was stable and yielded a standard deviation (*SD*) below 0.1 per day between all time‐points. Exceptions were strain A under warming conditions and strain C under the present‐day conditions, which maintained a higher variability throughout four to five dilution cycles (*SD* = 0.12 and 0.14 per day, respectively). Nevertheless, throughout the experiment, both strains grew with a standard deviation below 0.06 per day. During the experimental phase, the mean standard deviation of all strains was 0.04 per day. Since the required duration for acclimation by this definition is strain‐specific, experimental incubations took place within different overlapping timeslots. Although it would have been preferable to use a nonsequential design for incubation experiments, time blocking is unlikely to have affected results of this experiment, as growth rates were steady through time. In fact, we verified that monoculture growth rates did not vary over time by testing the effect of time on growth rates in all single strain culture growth curves during the experimental phase (three‐way ANOVA of factors strain, treatment, and time with factor time having no significant impact; *df* = 4, *F* = 1.3, *p* = 0.24).

Each treatment was conducted in independent biological triplicates for each strain, except for strain A (*n* = 2). All sampling and dilutions were conducted under sterile conditions using a laminar flow hood. Cells were cultivated in 0.2 μm sterile‐filtered Arctic seawater (salinity: 32) enriched with macronutrients (100 µmol/L NO_3_^−^, 6.2 µmol/L HPO_4_^2−^, 100 µmol/L SiOH_4_), vitamins, and trace metals according to f/2_R_ media (Guillard & Ryther, 1962). Cells were grown under continuous light with 51 ± 3 µmol photons m^−2^ s^−1^ using daylight lamps (Biolux T8, 6500K, Osram, Germany). Irradiance was adjusted with a black mesh fabric and measured in filled culturing bottles using a 4π sensor (Walz, Germany).

For the temperature treatments, target values of 2°C and 7°C were chosen to simulate the temperature cells are presently experiencing during spring and summer in Kongsfjord (Hegseth et al., 2019) as well as current and expected future mean spring bloom temperatures (AMAP, 2013; Beszczynska‐Möller, Fahrbach, Schauer, & Hansen, 2012). Experiments were performed in a temperature‐controlled 2°C room, with bottles immersed in water‐filled aquaria for additional temperature stability. 7°C treatments were established by additional heating of the aquaria by immersion thermostats (Corio CD, Julabo, Germany). Continuous surveillance with a temperature logger (Almemo 2890, Ahlborn, Germany) ensured temperature stability at 2 ± 0.17°C and 7 ± 0.06°C.

The multistrain cultures were assembled from identical cell concentrations of each single‐strain culture that had been previously acclimated to the respective growth conditions. These multistrain cultures were then incubated in two treatments (“present‐day”: 2°C and 400 µatm; “future”: 7°C and 1,200 µatm) with *n* = 3 and 4 replicate bottles, respectively. They were exposed to the same experimental setup as the single‐strain incubations with cell numbers ranging from 300 to 9,000 cells/ml. All replicates of the multistrain cultures were grown in parallel for 12 days (~13–14 generations) and diluted twice to 300 cells/ml (days 4 and 8) in order to ensure that carbonate chemistry and nutrients remained stable over the experiment.

### 2.3 Carbonate chemistry

Target pCO_2_ levels were established by continuous aeration with a gas flow rate of ~170 ml/min. The appropriately mixed air was delivered through sterile 0.2 µm air filters (Midisart 2000, Sartorius Stedim, Germany) provided by a custom‐built gas mixing system (see Hoppe, Holtz, Trimborn, & Rost, 2015). Before inoculation and each dilution, seawater was equilibrated (≥24 hr) to the treatment pCO_2_ at treatment temperature.

Total alkalinity (TA) samples of each culture, as well as of control bottles containing sterile medium, were taken during the final sampling. TA samples were 0.7 μm‐filtered (GF/F, Whatman, UK) and stored in 250 ml borosilicate bottles at 3°C until analysis. TA was determined by duplicate potentiometric titrations (Brewer, Bradshaw, & Williams, 1986) using a TitroLine alpha plus autosampler (Schott Instruments, Germany) and corrected using Certified Reference Materials supplied by A. Dickson (Scripps Institution of Oceanography, USA). Stability of carbonate chemistry was ensured by regular measurements of pH throughout the incubations using a three‐point calibrated potentiometric glass reference electrode (Aquatrode plus Pt1000; Metrohm, Switzerland). Values were corrected for temperature variation using the program CO_2_sys (Pierrot, Lewis, & Wallace, 2006) with dissociation constants of carbonic acid by Mehrbach, Culberson, Hawley, and Pytkowicz (1973), refitted by Dickson and Millero (1987). Following Hoppe, Langer, Rokitta, Wolf‐Gladrow, and Rost (2012), calculations of the full carbonate system on the final day of incubation were performed in the same program based on the measurements of TA and pH (Table S2). Deviations in calculated pCO_2_ of the incubations compared to abiotic control bottles were ≤7% in all treatments (except for strain C in the present‐day conditions with −18%, Table S2).

### 2.4 Growth, production rates, and cellular composition

Cell densities were counted daily using a Coulter Multisizer III (Beckman‐Coulter, USA), where *T. hyalina* cells were quantified within a clear peak in the size range of 11–21 µm. Specific growth rate constants µ (per day) were calculated by an exponential fit through measured cell numbers for each time point according to the formula:

(1) $$\mu =\frac{\ln N_{t}-\ln N_{0}}{\Delta t}$$

where *N_t_* refers to cell density at time *t*, *N*_0_ to the initial cell density, and ∆*t* to the passed time (in days) since the start of the incubation. Monoculture growth rate constants were based on at least two consecutive dilution cycles for each culture. Specific growth rate constant *µ* was converted into division rate *k* (i.e., divisions/day) by dividing *µ* by ln(2). Growth rate constants of the multistrain cultures were calculated for the last dilution cycle only (second dilution until final time‐point) since this was most comparable to acclimated state in terms of the time spent under a given set of conditions.

At the end of each experimental incubation, filter samples were taken to measure several cellular traits. For particulate organic carbon (POC) and nitrogen (PON), cells were filtered onto precombusted (15 hr, 500°C) glass fiber filters (GF/F, 0.7 µm nominal pore size; Whatman, UK) and stored at −20°C. Filters were soaked with HCl (200 µl, 0.2 M) to remove inorganic carbon and dried overnight at 60°C before POC analysis was performed, using a gas chromatograph CHNS‐O elemental analyzer (Euro EA 3,000; HEKAtech). POC values were blank corrected by the measurements of filters taken from pure medium. Daily production rates of POC were obtained by the multiplication of the respective elemental quota with corresponding division rates *k*.

Chlorophyll (Chl) *a* samples were filtered on GF/F filters, shock‐frozen in liquid nitrogen, and stored at −80°C. For analysis, filters were shredded in acetone (70%) with glass beads (0.5–1 mm diameter) in a homogenizer (Precellys Evolution, Bertin Technologies, France). After overnight extraction at 4°C, Chl *a* was measured fluorometrically (TD‐700; Turner Designs), including an acidification step (1 M HCl) to determine phaeopigments (Knap, Michaels, Close, Ducklow, & Dickson, 1996).

### 2.5 Variable Chl *a* fluorescence

Variable Chl *a* fluorescence of photosystem II was measured on the mixed culture experiment as well as the “present‐day” (2°C 400 µtm) and “future” (7°C 1,200 µatm) treatments of the single strain incubations using a fast repetition rate fluorometer (FRRf, FastOcean PTX; Chelsea Technologies, UK) in combination with a FastAct Laboratory system (Chelsea Technologies). Photosynthesis–irradiance (PI) curves were fitted according to Webb, Newton, and Starr (1974) and yielded estimates of maximum light‐use efficiency (α) and maximum absolute electron transport rate (ETR) through photosystem II (ETR_max_) as well as at the irradiance of growth conditions (in situ ETR). All measurements (*n* = 3–4) were conducted at the respective treatment temperature. Instrument settings as well as data processing and fitting were performed as described in Hoppe, Flintrop, and Rost (2018).

### 2.6 DNA sampling and extraction of multistrain cultures for microsatellite analysis

For a relative quantitative determination of genotype composition in the multistrain experiment, DNA samples were taken from each replicate at the time of every dilution and the final time point. Cultures were well mixed before 160–250 ml samples of each bottle were filtered on PC filters (Whatman Nucleopore), which were immediately added to vials containing extraction buffer and stored at −80°C. All multistrain DNA was extracted using the NucleoSpin Soil extraction kit (Macharey‐Nagel GmbH, Germany) while monocultures for microsatellite characterization were extracted with the NucleoSpin Plant II kit (Macharey‐Nagel GmbH), both according to the manufacturer's instructions with an additional cell disruption step in a cell homogenizer (Fast Prep FP120; Thermo Fisher, USA).

### 2.7 Allele‐specific quantitative PCR

The experiment described here was preceded by the development of six new microsatellite primers for *T. hyalina*; technical details can be found in the supplements of this article. In order to follow the genotype composition throughout the multistrain experiment, we modified a method described by Meyer, Ellner, Hairston, Jones, and Yoshida (2006) and John et al. (2015) as allele‐specific quantitative PCR (asqPCR). Five of the six strains of *T. hyalina* used in the multistrain experiment had at least one allele of unique size in one of the microsatellite loci ThKF3 or ThKF7. The only strain without a unique allele was strain A, which shared its homozygous allele of locus ThKF3 only with strain B (homozygous as well). However, this could be easily resolved since the abundance of strain B could be reliably determined from its two unique alleles in locus ThKF7. Accordingly, strain‐specific amplicons derived by PCR from multistrain DNA templates of filter samples as described above could be distinguished and their relative abundances quantified by asqPCR.

Relative abundances of the different strains were calculated from the peak area of the specific allele, that is, the sum of fluorescence signal from a strain‐specific allele, relative to total peak area measured. Total peak area was calculated for each sample as the sum of all peak area values minus the values of all stutter factors (*sf*, see below) taking results from linearity tests (see below) into account. For those genotypes that were homozygous in their specific allele, the according value was multiplied by 0.5. For the calculation of relative contributions of each allele, the two factors were also taken into account as described below.

#### 2.7.1 Stutter factor

Alleles of primer ThKF3 produced reliable stutter peaks at −1 and −3 base pair lengths from the main peak, which were correlated with the main peak area by a factor dependent on allele size. The stutter factor was established for each allele of locus ThKF3 based on the mean ratio of stutter versus allele peak of 120 monoculture DNA samples analyzed beforehand. In order to correct for the contribution of the stutter peaks of a larger allele to the area of a shorter allele, an allele‐specific stutter factor (*sf*) was multiplied with the peak area of the intruding larger allele. This value was then subtracted from the peak area value of the shorter allele. The amount of area “lost” was then added to the larger allele. Since primer ThKF7 did not produce any stutter peaks, the *sf* was here set to 0 for all its alleles.

#### 2.7.2 Linearity factor

The linear relationship between frequencies calculated from asqPCR assays and actual genotype frequencies was validated with standard curves derived from manual DNA mixes for both primers ThKF3 and ThKF7. We analyzed samples with relative contributions of each of the six strains at 0%, 5%, 10%, 16%, 25%, 33%, 50%, and 100%, which were added to a master‐mix of the remaining five at equal contributions. By linear regression, we could show that the relative contribution of an allele's peak area was directly proportional to the actual contribution of the respective cells' DNA in the mixture (Figure S1). Regression coefficients were measured in all cases with *r* ^2^ >0.99. The regression slope of each allele multiplied by 2 (to account for heterozygosity) was then used as the linearity factor (lf) for correction (0.8–1.0). In order to assess possible aberrations in extraction efficiencies of the different strains or alleles, we also tested the entire process from extraction to final relative contribution on artificial mixtures containing an equal number of cells of the six strains (as determined by a Coulter counter). Since the calculated contributions only deviated between 1% and 3% from the predicted values for each strain, we judged this error to be negligible. Accordingly, each allele frequency was calculated by:

(2) $$rF_{x}=\frac{\left(A_{x}*\left(1+sf_{x}\right)-\left(A_{x+3\text{bp}}*sf_{x+3\text{bp}}\right)\right)*lf_{x}}{\mathrm{tA}}$$

where *rF_x_* is the relative allele frequency of allele *x*, *A* is the measured peak area of allele *x* (or *x* + 3bp, i.e., the allele 3 base pairs upstream of *x*). *sf_x_* and *lf_x_* refer to the specific stutter and linearity factor for each allele, respectively. *tA* is the total peak area of a sample and was calculated as the sum of all corrected allele peaks.

### 2.8 Calculations and statistical analysis

For the monocultures, one objective of this study was to understand how trait responses (growth rates, cellular quota, and derived ratios) to environmental conditions (temperature and pCO_2_) varied between genotypes. To this end, we calculated the effect size as the raw mean difference with pooled standard deviations (following Borenstein, Hedges, Higgins, & Rothstein, 2009) of specific growth rates for the future and warming treatments compared to the control (present‐day) treatment for each strain. In addition, we calculated the raw mean deviation of single‐strain and multistrain culture growth rates relative the respective mean growth rate of all monoculture strains for each treatment. We also calculated the standardized effect size of both the treatment effect and the strain difference by dividing the raw mean difference by the pooled standard deviation. We used two‐way ANOVAs to test how each trait was affected by the identity of the strain (six strains = six levels) and by the environmental treatment (present‐day, warming, future = three levels) as well as by their interaction (strain × treatment) using the software R (vers. 3.1.1, 2014, R Foundation for Statistical Computing, Austria). Data were tested for normality (Shapiro–Wilk test) and homogeneity (Levene's test). Because of deviations from normal distribution, POC quota, POC production, Chl *a*:POC ratios, alpha, and ETR_max_ were log‐transformed prior to analysis. Since it is challenging to visualize patterns present in all measured traits across two treatments in six strains (Table S1), an additional principle component analysis was run with measured growth rates, cellular quotas, and ratios of each strain in monoculture as well as the multistrain culture for the present‐day and future treatment in the software r.

Differences between bulk responses in the multistrain cultures in the two environments (present‐day and future) were tested for each trait by one‐way ANOVAs after testing normality (Shapiro–Wilk test) and homogeneity (Levene's test). The number of generations (in the multistrain cultures) was calculated from the number of days of incubation and the bulk division rate (*k*, per day) of the cultures.

A second objective of the study was to understand how growth in monoculture and the composition of multistrain cultures are related. The predicted genotypic composition of the multistrain culture was calculated based on the specific growth rates of each strain in monoculture over the same time frame and dilution conditions as the experimental multistrain incubations. The standard deviation of growth rates for each strain in monoculture was used to calculate uncertainties in these predictions according to the law of propagation of uncertainties. Observed mean genotypic composition as well as standard deviation was calculated from the biological replicates of multistrain cultures (present‐day *n* = 3, future *n* = 4). Predicted and observed contribution of each strain to the final genotypic composition of the multistrain cultures was compared by Pearson's correlation coefficient (*R*). As a measure of diversity of the multistrain cultures, Pielou's evenness index (Pielou, 1966) was calculated from the observed relative contributions of each strain to the final genotypic composition of each replicate bottle as well as from the predicted contributions derived from monoculture growth rates.

All predicted bulk properties of the multistrain cultures were calculated from each strain's observed frequency in the multistrain culture, its cell properties measured in monoculture, and total cell abundance in the multistrain culture. Predicted and observed bulk responses of the multistrain cultures for each cell trait were compared using one‐way ANOVAs (as above). Observed growth rates in multistrain cultures were calculated for each strain based on its relative allele contribution (converted to cell number as fraction of total cell count) between the last dilution and the final time point of the experiment. The raw mean differences and standardized effect sizes of growth rate for each strain in mono‐ versus multistrain culture were calculated in the same way as above (following Borenstein et al., 2009) for the present‐day and future treatments.

## 3 RESULTS

### 3.1 Physiological responses of monocultures and multistrain cultures

**Figure 1:**
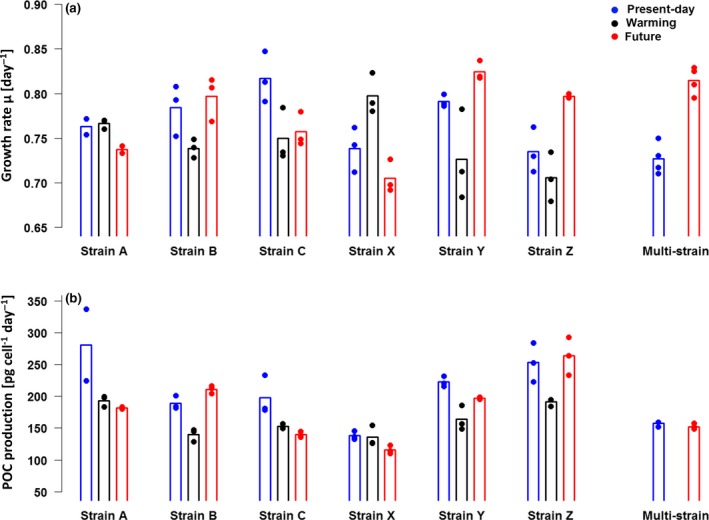
Intraspecific differences in growth and productivity under climate change treatments (temperature and pCO_2_). (a) Specific growth rates and (b) POC production of the monocultures and the multistrain culture in the 3 treatments (present‐day: blue, warming: black, future: red). Dots signify the value of the biological replicates, bars their respective mean

**Figure 2:**
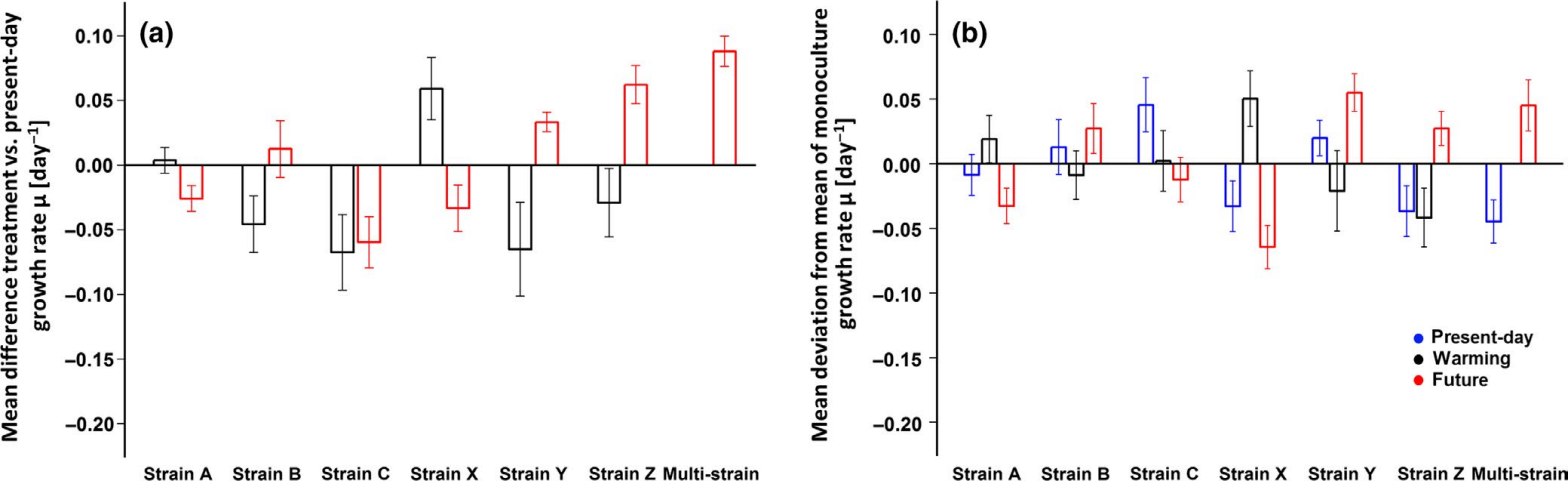
Differences in specific growth rate caused by treatment and by strain differences are comparable in scale. (a) Effect size as the raw mean difference ± pooled standard deviation of specific growth rates for the future and warming treatments compared to the control (present‐day) treatment for each strain. (b) Effect size as the raw mean deviation ± standard deviation of single‐strain and multistrain culture growth rates relative the respective mean growth rate of all monoculture strains for each treatment (present‐day: blue, warming: black, future: red)

All responses of monocultures were repeatable within strains but highly variable between them (Figures 1 and 2, Table S1). In all three environmental treatments (present‐day, warming, and future), the majority of strains exhibited different phenotypic traits (e.g., for growth, see Figure 2b). Although treatment effects were often pronounced within one strain, their direction and magnitude differed among strains (e.g., for growth, see Figure 2a). For instance, elevated temperature and pCO_2_ (future vs. present‐day treatment) impacted growth rate positively for strains Y and Z (by +4% and +8%), but negatively for strains A, C, and X (by −3% to −7%), and had no effect on growth rate for strain B (Figures 1a and 2a). POC production under high temperature and pCO_2_ was elevated in strain B (+11%), unchanged in strain Z, and lower in all other strains (−11% to −29%; Figure 1b). Elevated temperature alone increased growth rate relative to the present‐day treatment in only one strain (strain X by 8%), while it slowed growth in three strains (strains B, C, and Y by −6% to −8%) and had no effect in two strains (strains A and Z; Figures 1a and 2a). POC production was either not affected by warming (strain X) or lower under these conditions (−2% to −26%; Figure 1b). Because of this variability in responses between strains, the overall mean of all strains did not differ significantly between environmental treatments for most traits (e.g., µ, per day: present‐day: 0.77 ± 0.03, future: 0.75 ± 0.03, warming: 0.77 ± 0.01, Table S1). Still, in all tested traits except ETR_max_, both strain and treatment had statistically significant effects on trait values (two‐way ANOVAs, Table S3a). Since the responses to environmental treatments varied between strains, the interactive term of treatment and strain had the largest statistical effect on the growth responses (Table S3a). The standardized effect sizes of environmental treatment and strain differences were on a similar scale in the majority of cases and are shown in Table S4.

Responses of the multistrain cultures to the environmental treatments varied less across biological replicates than the monoculture replicates for most of the traits measured (Figure 1, Table S1). In the multistrain cultures, growth rate increased significantly in the future environmental treatment (Figure 1a; one‐way‐ANOVA: *F* = 62.7, *p* < 0.001, Table S3b), while POC quota decreased significantly under the same conditions (Table S1; one‐way ANOVA: *F* = 84.0, *p* < 0.001, Table S3b), causing POC production to stay constant in the two environmental treatment (Figure 1b; one‐way ANOVA, *F* = 3.99, *p* = 0.09, Table S3b). Notably, POC production of all multistrain cultures resembled those rates of the least productive monocultures (Figure 1b). Differences in photophysiological traits (alpha, ETR_max_, and in situ ETRs) between multistrain cultures in the two treatments were not significant (Table S3b).

### 3.2 Microsatellite locus characteristics and genotypic composition of multistrain cultures

The six loci used in this study were found to be differently polymorphic, resolving 4–24 alleles across all samples (Table 1). Excluding stutter peaks, loci reliably yielded one or two peaks for each genotype, implying successful isolation and establishment of monocultures of our diploid organism. From repeated amplification of identical genotype DNA, we established a technical error rate of allele identification of 2.1%. Several DNA templates of closely related species of the same origin (*T. gravida*, *T. nordenskoeldii*) did not yield any PCR products, indicating that cross‐amplification between species is unlikely here. Very low numbers of null alleles can be assumed, since all 365 strain samples showed amplification of one or two alleles and expected and observed heterozygosity showed high similarity for most loci (except in loci ThKF2 and ThKF6). While some loci tested positively for significant linkage disequilibrium (LD), the reciprocal combinations of them were not (e.g., LD was found in loci ThKF1 and 2 as well as ThKF1 and 3, but not in ThKF2 and 3).

**Table 1:** Properties of six microsatellite loci and their respective primers. Measures of observed and expected heterozygosity (H_O_ and H_E_) and linkage disequilibrium (+ indicates significant, ‐ no linkage equilibrium, * denotes not applicable) are based on the analysis of *n* = 364 single‐genotype samples

	Repeat pattern	Size range (bp)	Primer sequence fwd	Primer sequence rev	Color tag	Multiplex	No. of alleles	H_O_	H_E_	p H_0_/H_E_	Linkage disequilibrium						
											Locus	1	2	3	4	6	7
ThKF1	CTG	248–257	TCGTATGGCTGCCATGAGAAG	GTAACTGCTGGGACGACCAC	HEX	No	4	0.65	0.67	0.261	**1**	*	+	+	‐	‐	‐
ThKF2	CA	247–259	AATTTGGAAGCCGCCGTAGA	GGGTCGGAGAGTTTGTTGCA	AT	No	7	0.58	0.65	0.001	**2**	+	*	‐	‐	‐	‐
ThKF3	TCA	187–264	TCGCTGTCCTCGGTTTCAC	CAATGATGAGGTCCGGCGAT	FAM	No	24	0.84	0.85	0.051	**3**	+	‐	*	‐	‐	+
ThKF4	TTG	246–258	GGAGGAAAAACAACCGTTTGCT	TACAGGCCTTCCTTGCATGC	HEX	Multi#1	5	0.48	0.48	0.882	**4**	‐	‐	‐	*	‐	+
ThKF6	AAGTGA	229–247	AAATCCGCAGCCGAGAACAT	GAGAAGAGTCGCGCAGGATT	FAM	Multi#1	5	0.57	0.65	0.001	**6**	‐	‐	‐	‐	*	+
ThKF7	ACCAGC	215–290	ATTCCCATAGTCTCCCGACAGA	GGGGAGATCGTGATGCCTTC	FAM	Multi#2	14	0.80	0.84	0.485	**7**	‐	‐	+	+	+	*

**Figure 3:**
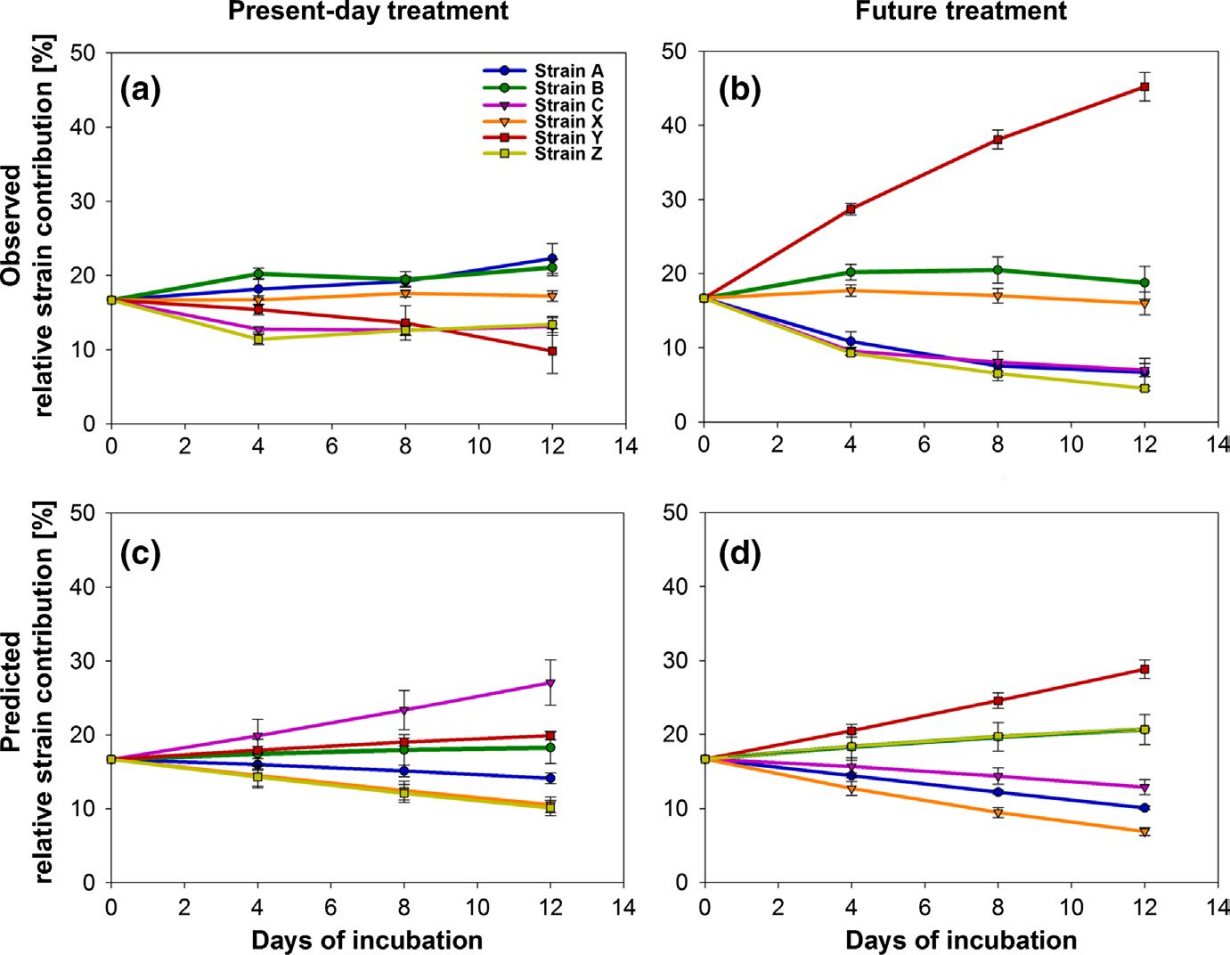
Genotype composition in the multistrain culture expressed as their relative contribution to the population (%) as measured via asqPCR (a, b) and predicted from monoculture growth rates (c, d) in the present‐day and the future treatment over the course of the experiment (13‐14 generations). Error bars in the observed measurements (a, b) denote standard deviations of the four biological replicates. Error bars in the predicted composition (c, d) show propagated uncertainties derived from standard deviations of specific growth rates in monoculture

Through asqPCR using our microsatellites, we followed the dynamics of change in relative strain abundances in the multistrain populations using filter samples taken at three time‐points (t1, t2, tfin). Previously, this method has only been used to quantify the relative abundance of pairs of genotypes (John et al., 2015; Minter, Lowe, Brockhurst, & Watts, 2015; Sildever, Sefbom, Lips, & Godhe, 2016). Here, we extended this method to monitor the relative abundances of six genotypes in artificial assemblages. The strain frequency measurements were highly repeatable across all replicate incubations, which are reflected in the small standard deviations in Figure 3a,b. This indicates that the dynamics of competition between strains were consistent across multistrain cultures and mirrors their repeatable physiological bulk responses (Table S1). In the present‐day treatment, strain frequencies showed only small temporal changes throughout the experiment (~13 generations), except for a slight decrease in the frequency of strain Y. In the future treatment, relative strain abundances changed substantially and resulted in a clear dominance of strain Y (43%–47%) within the same timeframe. As a result, the Pielou's evenness in the two environmental treatment differed significantly (one‐way ANOVA: *F* = 100, *p* < 0.01; Table 2, Table S3b). No strain extinctions in the mixed cultures were observed in the timeframe of the experiment.

**Table 2:**
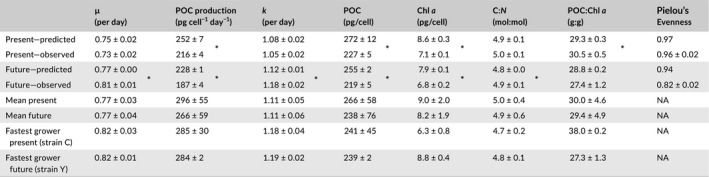
Predicted and observed bulk responses in multistrain incubation ± standard deviation of biological replicates. *Significant difference between the predicted and observed value (one‐way‐ANOVA, α = 0.05, see Table S3c), except for Pielou's evenness which could not be tested. Predicted numbers were calculated from the measured strain composition, assuming their respective values in monoculture. For reference, the mean of all monocultures as well as the properties of the fastest growing strain in monoculture are also depicted for both treatments

### 3.3 Prediction of multistrain cultures from monoculture responses

The “predicted” strain composition in the multistrain culture was based on the growth rate constants measured in the monocultures under different environmental treatments, and therefore, each strain's relative abundance was predicted to change linearly of over time (Figure 3c,d). This resulted in the expectation that strain frequencies would differ between environmental treatments, but that diversity would be approximately the same over environmental treatments (Pielou's evenness present‐day: 0.97 and future: 0.94; Figure 3c,d, Table 2). In contrast, the measured strain composition of the assemblages grown under the present‐day environmental conditions changed slightly less than predicted (Figure 3a vs. 3c) and strains remained close to their original inoculation frequencies (16.6%) throughout the experiment. Under future environmental conditions, the strain that had been growing fastest in monoculture under those same conditions (strain Y) indeed dominated the final assemblage, but had a higher final frequency than predicted (observed contribution final time point: 45% vs. predicted 28%, Figure 3b vs. 3d and Table S1). The predicted and observed Pielou's evenness differed strongly in the future, but not the present‐day treatment (Table 2). Linear regressions between predicted and observed strain frequencies showed that in the present‐day treatment, the monoculture growth rates were a poor predictor of the strain composition of the multistrain cultures (*R* = −0.33, Figure S3a). In the future treatment, this correlation was slightly better (*R* = 0.67, Figure S3b), even though this was mainly driven by the correct prediction of strain Y becoming the dominant genotype in the multistrain cultures.

**Figure 4:**
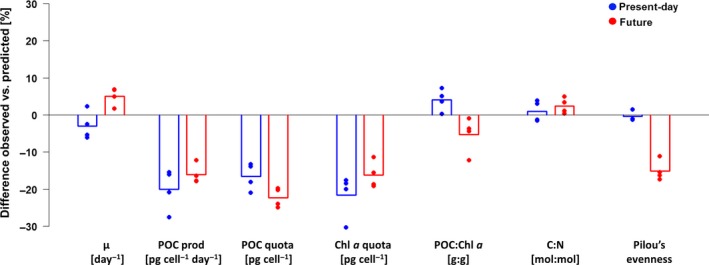
Raw difference of observed bulk physiological responses of the multistrain culture compared to the predicted value as calculated from monoculture responses considering the observed final strain composition in the two tested environmental treatments (c.f. Table 2). Dots signify the value of the biological replicates, bars their respective mean (present‐day: blue, future: red)

**Figure 5:**
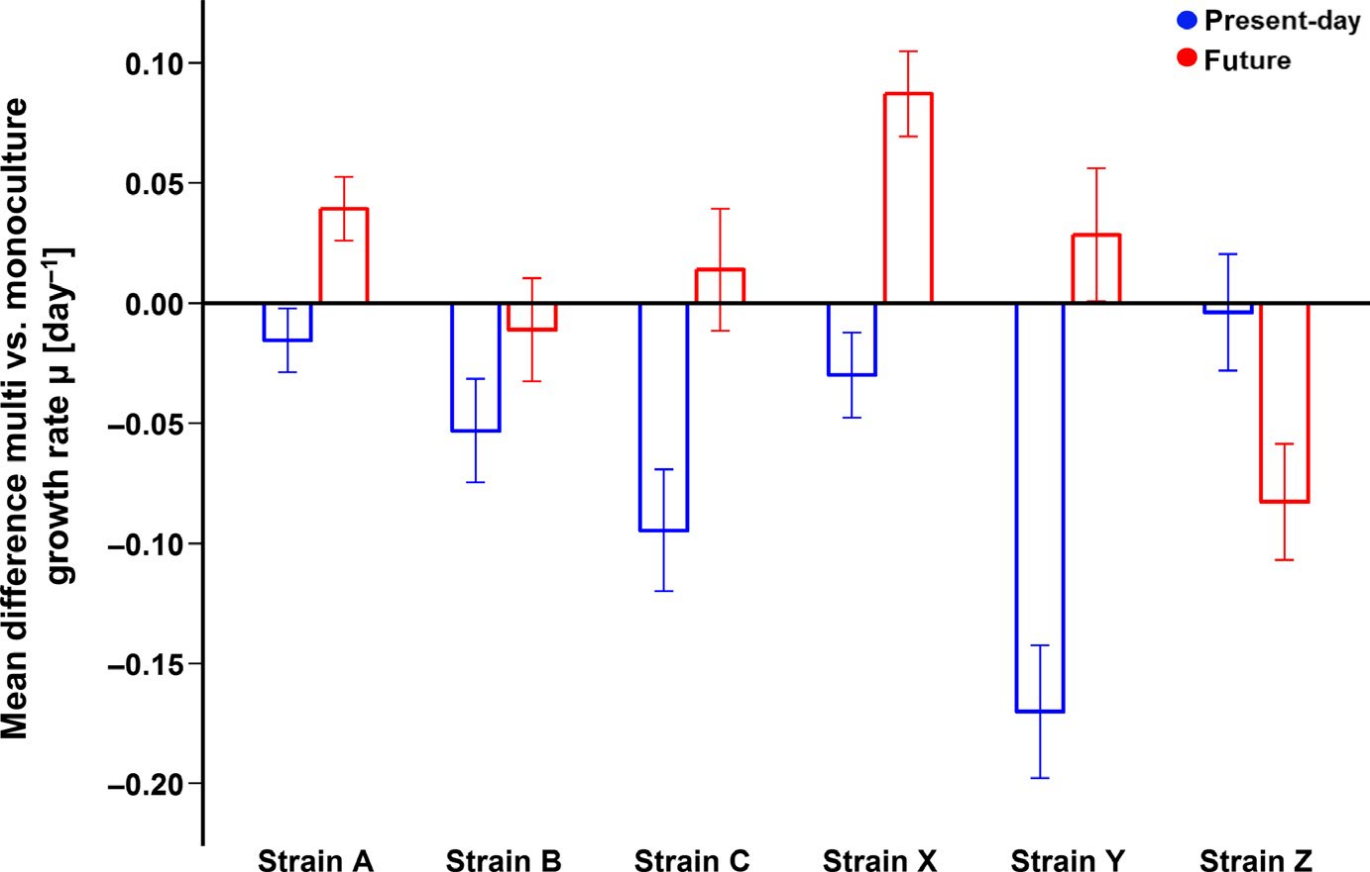
Effect of diversity on specific growth rates. Raw mean difference and pooled standard deviation of each strain's growth rate in the multistrain cultures (calculated from measured allele contributions over time) compared to the ones measured in monoculture. Since the diversity level was the only component changed, this represents the effect of diversity or genotype interactions

The predicted bulk responses of the multistrain cultures (calculated based on strain composition and monoculture quota) are referred to as “predicted values” here. A comparison of these predicted and observed values can be found in Table 2 and Figure 4: for the majority of traits, the predicted values were significantly different from the observed ones (one‐way ANOVAs, Table S3c). Similarly, the mean of all monoculture traits as well as the traits of the fastest growing strain deviated considerably from the observed multistrain values (Table 2). Predicted bulk growth rates were slightly but not significantly higher than the measured values in the present‐day, but significantly lower than those measured in the future treatment (Table 2). Calculated for each strain individually, in both environmental treatments, most observed growth rates differed strongly in mono‐ compared to multistrain cultures (Figure 5). Observed POC and Chl *a* quota in all multistrain cultures were much lower than predicted, causing production rates to be strongly overestimated, despite increased growth rates in the future treatment (Figure 4, Table 2).

## 4 DISCUSSION

### 4.1 Wide and diverse temperature and CO_2_ niches within the same population

All six strains in this study grew well in the three environmental treatments, that is, the ambient conditions (present‐day), warming alone (warming), and warming in combination with elevated pCO_2_ (future). If the plastic responses observed here can be maintained over time, all strains appear to have a sufficiently wide fundamental ecological niche to sustain growth and productivity under conditions anticipated for the coming decades. Our results are in line with reaction norms of *T. hyalina* strains isolated 2 years earlier from the same location (Wolf et al., 2018). Both datasets also show that underlying reaction norms differ between strains, which may be due to different physiological fine‐tuning. The raw mean differences in growth rate constants among strains varied by up to 0.1 per day (Figures 1a and 2b; standardized effect size, Table S4). While these differences in growth rate may appear small in absolute terms, the range of growth rates observed here within a single species is comparable with differences previously found between species (e.g., Pardew et al., 2018; Schlie & Karsten, 2016) and is certainly ecologically relevant (Schaum, Rost, Millar, & Collins, 2012) as is readily visible in the predicted population composition (Figure 3c,d).

Although most strains exhibited reproducible differences in trait values between environmental treatments (i.e., treatment effects; Figure 2a), the pooled mean trait values of all strains within each treatment were hardly affected (Table S1). This is because the environmental treatment effects on traits differed between strains in both magnitude and direction (Figure 2a). The growth responses among strains to high temperature and pCO_2_ (i.e., future vs. present‐day treatment) were especially diverse, with growth rate changes between −7% and +8% (Figures 1a and 2a). Elevated temperature alone (i.e., the warming treatment) often had a different effect on trait values than warming in combination with high pCO_2_: in contrast to usual expectations for cold‐adapted species (Eppley, 1972; Kremer, Thomas, & Litchman, 2017; Thomas, Kremer, Klausmeier, & Litchman, 2012), three of six strains grew slower and only one faster at 7°C compared to 2°C under the present pCO_2_ (Figures 1a and 2a). While POC production did not show a uniform pattern across strains within environmental treatments, the majority of strains decreased POC production rate in the future treatment, with the decrease being even more marked under warming alone (Figure 1b).

The raw mean growth differences between strains were in the same range as those of the environmental treatments (Figure 2a vs. 2b), which is also visible in the similarity of standardized effect sizes (Table S4). This illustrates that intraspecific phenotypic differences can equal or surpass the influence of projected future environmental change on trait values. The relevance of these intraspecific differences is supported by the fact that strain identity and their interaction had significant effects in all measured cell properties and all three environmental treatments (two‐way ANOVAs; Table S3a).

To date, results of experiments with natural assemblages carried out over tens of generations have often been interpreted to be caused by selection for individuals with different response optima from the standing diversity (Collins, Rost, & Rynearson, 2014; Scheinin, Riebesell, Rynearson, Lohbeck, & Collins, 2015; Wolf et al., 2018). This is partly because even if novel mutations do provide beneficial alleles, within such relatively short experiments, they would not have sufficient time to reach high frequencies unless they fall far outside the range of the present standing variation. In this study, two of three strains from each of the two isolation backgrounds of the preceding natural community incubation grew faster in the treatment most resembling their origin (i.e., strains A, B, C from the present‐day vs. strains X, Y, Z from future conditions; Figure 1b). A similar pattern emerges when taking all measured traits into account (e.g., in a principal component analysis, Figure S2). This is only partly consistent with expected strain sorting according to abiotic conditions within the natural community incubation prior to isolation. Still, since six strains are a small sample size compared to the natural standing diversity and as the responses are not uniform, this cannot clearly support or falsify the idea of intraspecific sorting in the community incubation as hypothesized in Hoppe, Wolf, et al. (2018).

Comparing the traits of all six strains growing in different environmental treatments, neither of the drivers had a consistently positive or negative effect (Figures 1 and 2a). Due to this complexity in physiological responses, we cannot expect to find a representative trend in reactions to warming and high pCO_2_ using a small number of strains, even if they originate from the same population. This suggests that the usual parameterizations of ecosystem models based on upscaling of physiological responses of single strains may not accurately project the properties of future populations, and that projecting the range of trait values available to a given phytoplankton functional type requires an accurate estimate of intraspecific trait variation. Furthermore, the differences in growth rate between strains show that there is a high potential for rapid intraspecific sorting and thus for rapid selection within a population. By applying allele‐specific quantitative PCR, to our knowledge for the first time in such a setup, we were able to follow strain sorting directly over short timescales and thus to resolve how this potential was realized in a simplified assemblage.

### 4.2 Rapid strain sorting under future but not under the present‐day conditions

As described in the introduction, several ways of predicting the genotypic composition and yield of multistrain cultures from its components in monoculture have been suggested. None of them fully explains our results as shown in Table 2. Under future conditions, we were able to partially predict the strain composition of the multistrain assemblages from growth rates in monoculture (Figure 3b,d; Figure S3b). In line with selection effects, here the multistrain growth rate also resembled that of the fastest growing strain in monoculture (Table 2) and indeed the fastest growing strain dominated after 14 generations with 43%–47% (strain Y). However, in this treatment, strain sorting was even more pronounced than anticipated based on predictions made from monoculture growth rates (Figure 3b vs. 3d). These rapid selection dynamics support the view that strain sorting by natural selection can indeed influence population composition and performance even on short timescales relevant for bloom dynamics (Godhe & Rynearson, 2017; Scheinin et al., 2015).

However, even in the presence of variation in strain growth rates in monoculture, such rapid sorting does not always occur, as was revealed in the multistrain incubations under the present‐day conditions (Figure 3a). Under those conditions, the fastest growing strain in monoculture failed to dominate the multistrain cultures, and bulk population growth instead resembled the lowest rate measured in the monocultures in the same environment (Figure 1a, Table S1). Here, changes in strain composition of the multistrain assemblage provided little evidence that growth in monoculture predicted strain growth rates in mixed culture (Figure S3a), and strain abundances diverged slightly less and with different strain proportions than predicted (Figure 3a vs. 3c). Hence, especially in the present‐day environment, strains responded strongly to the presence of other genotypes. Here, the different strains seemed to be roughly of equal fitness since most strains remained at rather constant frequencies. Only strain Y, which dominated the future treatment, slightly decreasing in cell abundance. This example suggests that there may be a trade‐off causing divergent competitive abilities under the two environmental treatments. In both treatments, strain sorting in the multistrain cultures showed different dynamics than those predicted from monoculture growth rates, which illustrates that strain‐specific growth rates appear to differ in multistrain assemblages compared to monoculture (Figure 5, Figure S3).

Bulk growth rates of the whole multistrain assemblage in the future environmental treatment were significantly higher than predicted from monoculture growth rates for the observed strain composition (Figure 4, Table 2). In the present‐day treatment, however, the population growth rate was similar to, but slightly below the predicted one. In both environmental treatments, POC production was far lower than any prediction based on monoculture traits (Figure 4, Table 2). The reduced POC productivity in multistrain cultures (Figures 1b and 4) does not support the idea that diverse communities are at least as productive as monocultures (Hector, 1998). In phytoplankton, however, the relationship between mono‐ and multistrain cultures has been studied mainly using population growth rates rather than productivity (e.g., Bell, 1991; Hattich et al., 2017). In studies that measure population growth rate, negative diversity effects have been described (Roger et al., 2012). In an experimental evolution study, Collins (2010) found that multistrain cultures had repeatedly lower yields than their constituent monocultures at the same abundance after adapting to elevated pCO_2_. This suggests that selection based on competition between genotypes may cause different outcomes than adaptive selection driven by the abiotic environment alone. It has also been proposed that cell division rates lower than the unevolved plastic response may be adaptive under long‐term CO_2_ enrichment, when the initial response to enrichment is to increase cell division rates (Collins, 2016; Schaum & Collins, 2014).

Despite strain‐specific treatment effects in monoculture and large differences in strain composition, POC production changed remarkably little across the environmental treatments in all multistrain cultures (Figure 1b). Interestingly, this stability is consistent with the concept of insurance effects (Yachi & Loreau, 1999) as well as with the primary production estimates of the community incubation the strains were originally isolated from, which were also largely insensitive to environmental treatments (Hoppe, Wolf, et al., 2018; data KFb). Thus, the same mechanisms stabilizing POC production in our simplified populations may have contributed to the compensation of CO_2_ effects in the natural assemblages, even though we cannot say to what extent. The stability of POC production in the multistrain cultures is an effect of the opposing trends of growth rate and POC quota in both environmental treatments. Hence, populations did not become more or less productive (which is also in line with the stable photophysiology; Tables S1 and S3d), but merely reallocated their energy budget toward faster division rates in the future and increased carbon storage in the present‐day treatment (cf. Behrenfeld, Halsey, & Milligan, 2008).

Considering the consistent differences in predicted and observed multistrain bulk trait values of POC and Chl *a* (Figure 4, Table 2) within both environmental treatments, we can conclude that strains must also change their cellular quota depending on whether they are growing alone or in a multistrain assemblage. This means that the strain composition and bulk traits of even a simplified population are not predictable from the strains' trait values in monoculture, even though it is reproducible for a given strain assemblage and environment. Since we controlled for confounding influences (e.g., all cultures were previously acclimated and remained in exponential growth under stable irradiances and nutrient‐replete conditions), the single difference between the mono‐ and multistrain cultures was their genotypic diversity. We therefore hypothesize that strains alter their phenotype in response not only to their physicochemical surroundings but also to their intraspecific context; the presence of other conspecific genotypes (i.e., diversity) may be a cryptic driver for trait responses that has often been neglected so far.

### 4.3 Diversity as an additional response driver

If the proximity of other conspecific strains acts as an additional driver, we should be able to quantify it by comparing the observed properties of the multistrain incubations with the predicted ones. Indeed, for most bulk traits, the effect of the presence of other conspecifics was reproducible and significant (Figure 4, Table 2). The scale and variability of this diversity effect on growth rate within and between strains were similar to that of altered temperature and pCO_2_ (cf. Figures 2 and 5, Table S4). Moreover, the resulting genotypic composition of populations was highly reproducible in all our incubations, a pattern that we also see in previous intraspecific competition experiments under a multitude of treatments (Bell, 1991; Collins, 2010; Lohbeck, Riebesell, & Reusch, 2012; Roger et al., 2012; Sjöqvist & Kremp, 2016). This suggests that differences in mono‐ and multistrain culture responses may be a definable eco‐evolutionary driver that we do not yet understand.

Biomass buildup and strain composition, being the final consequences of all drivers combined in a multistrain culture, may be understood as the result of an interplay of several selection pressures. Since the strongest drivers shape responses the most, they are usually considered the best predictor of how abiotic factors act as selective pressures on individual strains (Boyd et al., 2015; Brennan, Colegrave, & Collins, 2017). Therefore, the most successful strain in a selection environment is not necessarily adapted to be the fastest grower in a laboratory monoculture (Bach, Lohbeck, Reusch, & Riebesell, 2018; Schaum & Collins, 2014), but is determined by the strongest drivers in the fitness landscape of interest. Under the future environmental treatment, sorting in the multistrain culture was much better predicted by the monoculture responses than under the present‐day conditions (Figure S3: *R*: present‐day = −0.33, future = 0.67). This suggests that the effect of diversity was larger under the present‐day than under the future conditions for most strains (Figure 5). Assuming that the abiotic environment of elevated temperature and pCO_2_ exposed strains to stronger selection pressures than the present‐day treatment, where experimental conditions resembled the environmental history of the strains, we can make inferences on the role of diversity effects. In the future treatment, the abiotic treatment effects (Figures 1 and 2a) may have been more influential than the effect of intraspecific diversity (Figure 5). This could have caused our monoculture‐based predictions to be more accurate for the future conditions, while under the present‐day conditions, biological interactions may have had a larger impact (Figure 5), causing the selective outcome to be less predictable from monoculture responses.

The results of this study are consistent with organisms modulating their phenotype in response to the presence of other conspecific strains. A similar effect has been observed in incubations of a coccolithophore (Bach et al., 2018). There are numerous ideas for the underlying explanations of such diversity effects, and it is possible that they are caused by several interacting mechanisms at once, whose effects may add up or oppose each other. Explanations include direct and indirect competitive interactions (Collins, 2010), for example, by chemical cues, mutual facilitation between genotypes (John et al., 2015), nutrient partitioning (Vanelslander et al., 2009), or interactions with the prokaryotic microbiome (Amin et al., 2015; Camarena‐Gomez et al., 2018). However, direct evidence for such mechanisms in phytoplankton is rare and mainly descriptive (Brodie et al., 2017; Lima‐Mendez et al., 2015). In the future, we need to gain a mechanistic understanding of such effects, for example, whether they are explained by chemical cues or by more indirect competitive mechanisms.

### 4.4 Ecological implications

Our study suggests that intraspecific strain sorting may have a larger impact when environmental conditions differ more from the environmental history of populations. Thus, intraspecific strain sorting could buffer (or amplify) measurable effects at other levels of organization, such as species composition, productivity, and elemental stoichiometry (Hoppe, Wolf, et al., 2018). In the absence of rapid mutations, strain sorting in response to warming and acidification could lead to extinctions and decrease intraspecific diversity, which could in turn reduce the species' adaptive capability in the face of other pressures (e.g., nutrient limitation as the bloom enters a stationary phase). However, all existing evidence suggests that diatom populations are highly diverse (Godhe & Rynearson, 2017) and unlikely to be destabilized by moderate environmental shifts, especially in fluctuating environments (Gsell et al., 2012). Even in our comparably small assemblage of six strains, and despite considerable sorting in the future treatment, measures of diversity like Pielou's evenness index remained high until the end of the experiment (0.82, Table 2). However, to fully answer ecologically important questions about how intraspecific selection may alter the diversity and productivity of future phytoplankton populations, we need to move toward experimental setups with increasingly realistic diversity and environmental variability levels (Kroeker, Kordas, & Harley, 2017; Sjöqvist & Kremp, 2016). It will also be necessary to systematically understand the mechanisms by which microbes affect each other in diverse populations. This is particularly important as it is still challenging to resolve these processes in natural populations with commonly used methods, and intraspecific diversity is often too high to identify such patterns (e.g., Godhe et al., 2016; Ruggiero et al., 2017; Rynearson & Armbrust, 2005).

Several conclusions can be drawn from this study. We add evidence to the increasingly recognized view that individuals of the same population vary in their response strategies to elevated temperature and pCO_2_. At the same time, within our experimental climate change scenarios, even a low strain diversity buffered changes in the bulk productivity of the population. The extent to which such stability can be generalized needs to be investigated, also in the context of other stressors (e.g., light or nutrient limitation). The high resolution of the strain composition in our multistrain experiment reveals two novel findings: Firstly, different components of fitness seem to be under selection in different environments causing diverging selection dynamics and outcomes. Secondly, our data suggest that strains respond phenotypically to the presence of other conspecifics. In this case, phenotypic modulation appears to lead to trait changes that are on the same order as responses to our abiotic treatments. This provides further evidence that a rigorous method for upscaling single strain responses to populations requires a better understanding of the mechanisms shaping intraspecific selection. Evaluating genotypic diversity as an additional, potentially quantifiable driver may be a step toward making natural community responses more predictable from laboratory experiments.
