# Supplementary material for: Company matters: The presence of other genotypes alters traits and intraspecific selection in an Arctic diatom under climate change
Source: Glob Chang Biol. 2019 Jul 2;25(9):2869–84. doi: 10.1111/gcb.14675 (PMC6852494; doi:10.1111/gcb.14675)
Supplement: Supplementary file 1 [file GCB-25-2869-s001.docx]

**Supplement Material Wolf et al. “Company Matters”**

**Microsatellite development**

For the development of microsatellite primers, DNA was extracted from three exponentially growing axenic monocultures of *T. hyalina* (one isolated in 2014, two in 2016). DNA was sequenced (150 bp paired-end) on an Illumina NextSeq500 sequencer (Illumina, San Diego, USA). Illumina BCL files were converted to fastq files and de-multiplexed using bcl2fastq (v2.17, Illumina) with default settings. Sequence reads were trimmed and assembled with CLC Genomics Workbench v9 (CLC bio, Qiagen, Germany). Microsatellites were identified within those three genome assemblies and characterised in Phobos v3.3.12 (Mayer et al., 2010) using a maximum unit length of 10, maximal mismatch score of -4, maximal gap score of -4 and maximum score reduction of 3. For qualified tandem repeats, 300 bp of flanking region were exported together with the microsatellite. A second output file was generated in Phobos by masking all microsatellites in the genome assemblies. Subsequently, the two output files per genome assembly were compared using in-house scripts and manual inspections to obtain only those loci with at least 50 bp of flanking regions on each site, at least 50 bp between two microsatellites and a minimum normalized repeat length of 4. Shared loci among the three individuals were identified by mapping one genome assembly against the others using the mem module of bwa (Li & Durbin 2009). The output format was transformed with samtools (Li et al. 2009). Mapped microsatellites were filtered to include only those without alternative hits, without soft masking (meaning low alignment quality) and with mapQ values above 25. Microsatellites with indels or substitutions in the 50 bp flanking region were excluded. Nine microsatellites with differing tandem repeat length for all three individuals were thus identified.

The according microsatellite primers (Table 1) were tested on eight *T. hyalina* strains with and without fluorescent markers (FAM, HEX, AT), in single and multiplex conditions. The following optimized PCR conditions were applied for all primers with the Type-it Microsatellite PCR kit (Qiagen, according to providers instructions) in a thermal cycler (Mastercycler Nexus gradient, Germany): 5min at 94°C prior to 30 cycles of 30s at 94°C, 90s at 57°C, 40s at 72°C and a final elongation step at 72°C for 10 min.

For microsatellite application and fragment size analysis PCR products were diluted with nucleotide-free water at 1:45 (1:35 for Mutiplex-PCR). Subsequently, 1 µL was added to 15 µL of Hi-Di formamide (Applied Biosystems, Germany) and 0.3 µL of the size marker genescan-500 [ROX] (Applied Biosystems). Size analysis was performed by capillary electrophoresis on a 3130xl Genetic Analyzer (Applied Biosystems). Microsatellite alleles were scored using Genemapper (version 4, Applied Biosystems). The software Arlequin (version 3.5.2.2; Excoffier & Lischer, 2010) was used to test pairs of loci for linkage disequilibrium. The acquired loci were characterized in DNA of 364 samples of single strains from a *T. hyalina* population collected 2016 in Svalbard (Wolf et al., in prep.). Six differently polymorphic (4-24 alleles) loci were found to yield reliable results in *T. hyalina* (Table 1) and were named ‘ThKF’ for *T. hyalina* from Kongsfjord. In this study, two of them (primer ThKF3 and 7) were applied for allele-specific-quantitative PCR (John et al., 2015; Meyer et al., 2006).

**Figure S1**: Regressions of linearity test for asqPCR (2 examples) evaluating the allele frequencies measured in an artificial mixture of known DNA concentration from each strain: a) Regression of allele 208 with primer ThKF3 (unique for strain Y) b) Regression of allele 233 with primer ThKF7 (unique for strain B). Measured concentrations have a maximum of 50% because the strains contain the respective allele heterozygously. All alleles yielded a linear relationship with an R²-value > 0.99.

**Figure S2:** Principal Component Analysis (PCA) of the physiological responses of strains in monoculture and the multi-strain culture (Mix) in the present (blue) and the future (red) scenario. Analysis based on the parameters: µ, Chl *a* quota, POC quota, POC production, C:N ratio and Chl *a*:POC ratio. Two loose clusters appear in the present-day condition (containing strains B, C, X and A, Y, Z), while strains are more spread out in future treatment. While the multi-strain culture (Mix) of the present-day treatment is close to the center (i.e. the mean of strains), it deviates more from the center under future conditions. The two components depicted here explain 78% of the variance.


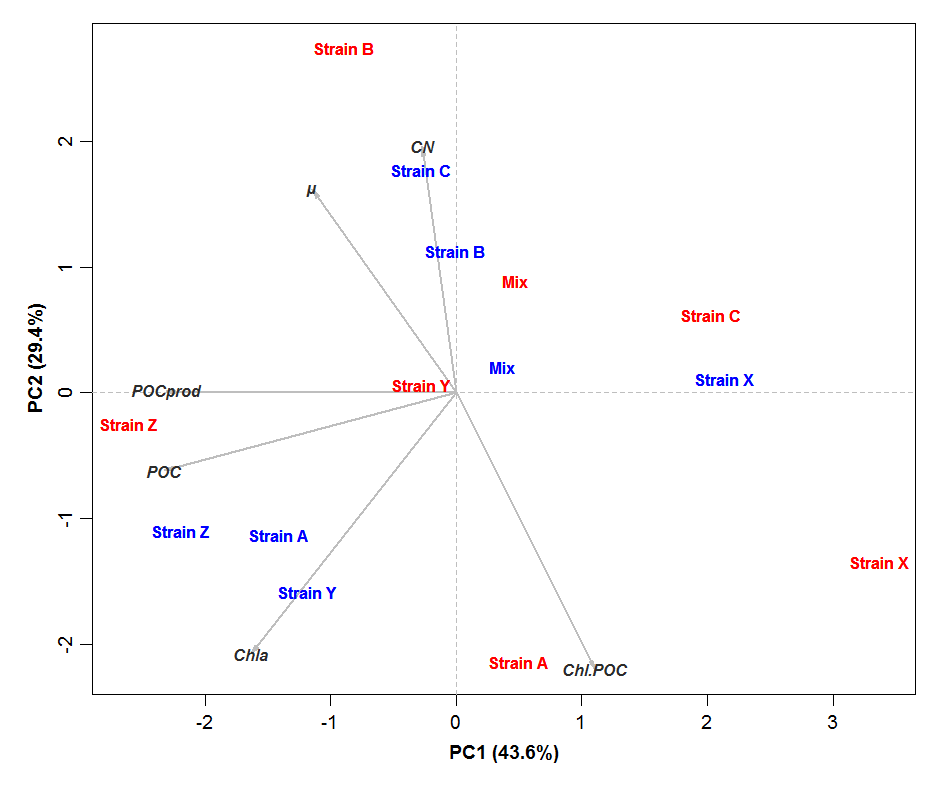


**Figure S3:** Correlation of predicted and observed contributions of each strain in the multi-strain incubations using Pearson’s R under the a) present-day (R= -0.33) and b) future scenario (R= 0.67).

**Pearson’s R=0.67**

**Pearson’s R=-0.33**

**Figure S4**: Stacked relative strain contribution in the multi-strain culture in % as observed via asqPCR and predicted from monoculture growth rates in the present-day and the future scenario.


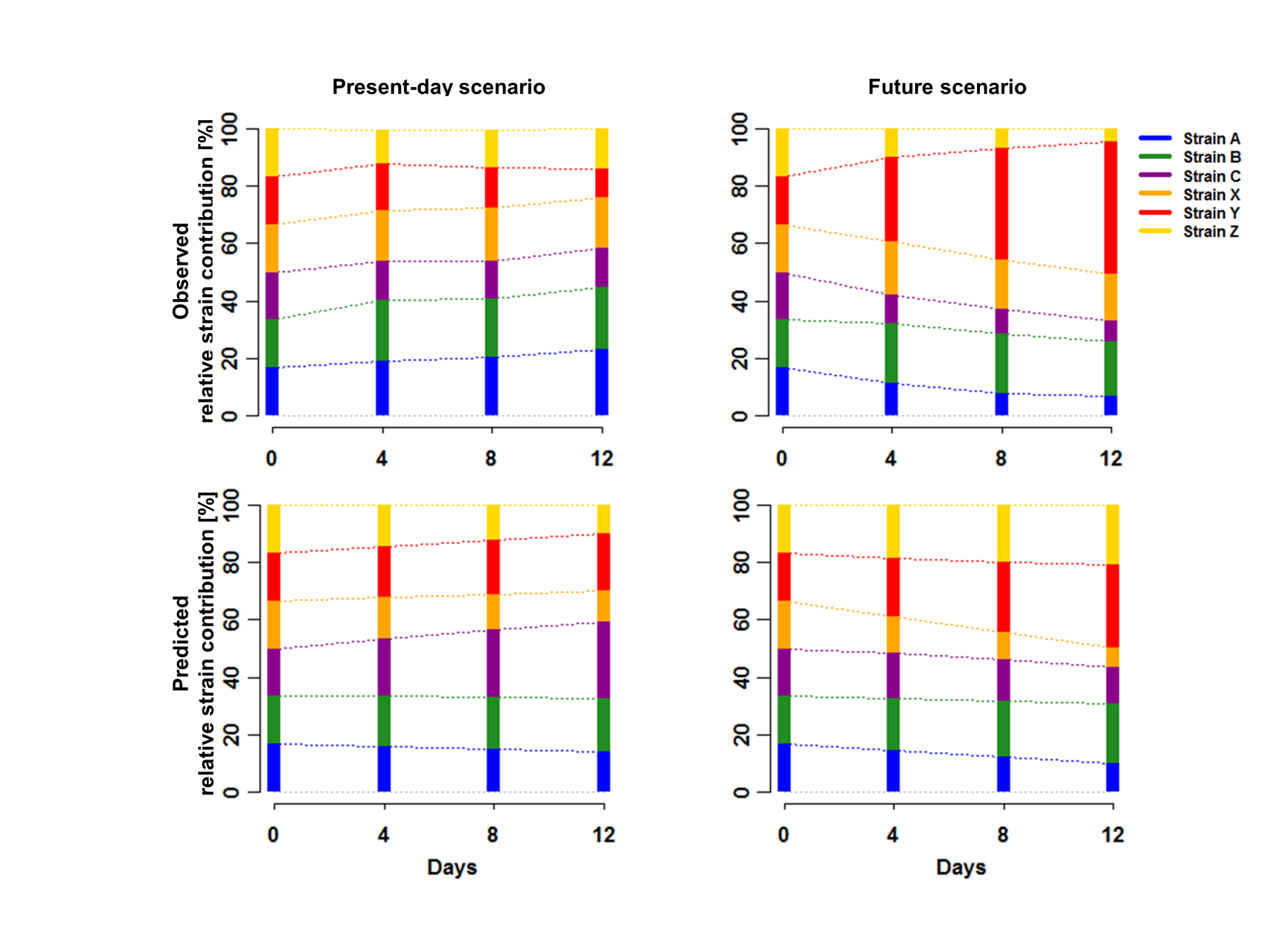


**Table S1:** Physiological strain responses in monoculture and bulk responses of multi-strain cultures as mean ± standard deviation. Relative contribution shows the observed relative frequency of each strain in the multi-strain incubation at the final time point of the experiment (13-14 generations) in percent. α refers to maximum light-use efficiency (initial slope α, mole^−^m^2^ (mol RCII)^−1^ (mol photons)^−1^), ETR_max_ describes the maximum absolute electron transport rate through photosystem II (mol e^−^(mol RCII)^−1^ s^−1^), in-situ ETR refers to the irradiance of the growth conditions. NA stands for not acquired or not applicable.

| **culture** | **treatment** | **µ**  **[day^-1^]** | **contribution [%]** | **k**  **[day^-1^]** | **POC**  **[pg cell^-1^]** | **POC production**  **[pg cell^-1^ day^-1^]** | **Chl*a***  **[pg cell^-1^]** | **C:N**  **[mol:mol]** | **POC:Chl*a* [g:g]** | **alpha** | **ETR_max_** | **In-situ ETR** |
| --- | --- | --- | --- | --- | --- | --- | --- | --- | --- | --- | --- | --- |
| Strain A | present | 0.76 ± 0.01 | 22 ± 2 | 1.10 ± 0.02 | 300 ± 14 | 330 ± 9 | 10.7 ± 0.2 | 4.9 ± 0.04 | 28.0 ± 1.7 | 1.37 ± 0.04 | 61.5 ± 6.8 | 45.0 ± 4.3 |
|  | future | 0.74 ± 0.01 | 7 ± 2 | 1.06 ± 0.01 | 246 ± 5 | 262 ± 3 | 10.6 ± 1.2 | 4.8 ± 0.01 | 23.4 ± 2.2 | 1.28 ± 0.07 | 72.1 ± 3.4 | 56.6 ± 5.4 |
|  | warming | 0.77 ± 0.01 | NA | 1.11 ± 0.01 | 252 ± 13 | 279 ± 13 | 9.2 ± 0.1 | 4.8 ± 0.2 | 27.5 ± 1.5 | NA | NA | NA |
| Strain B | present | 0.78 ± 0.03 | 21 ± 1 | 1.13 ± 0.04 | 241 ± 8 | 273 ± 15 | 7.9 ± 0.3 | 5.5 ± 0.7 | 30.6 ± 2.0 | 1.41 ± 0.04 | 60.9 ± 3.5 | 43.3 ± 3.4 |
|  | future | 0.8 ± 0.02 | 19 ± 2 | 1.15 ± 0.04 | 264 ± 1 | 304 ± 9 | 7.0 ± 0.1 | 6.0 ± 0.7 | 37.9 ± 0.6 | 1.32 ± 0.06 | 44.5 ± 1.4 | 33.7 ± 2.5 |
|  | warming | 0.74 ± 0.01 | NA | 1.07 ± 0.01 | 190 ± 14 | 202 ± 14 | 5.3 ± 0.5 | 4.5 ± 0.9 | 35.7 ± 3.2 | NA | NA | NA |
| Strain C | present | 0.82 ± 0.03 | 13 ± 1 | 1.18 ± 0.04 | 241 ± 30 | 285 ± 45 | 6.3 ± 0.8 | 4.7 ± 0.2 | 38.0 ± 0.2 | 1.26 ± 0.00 | 57.9 ± 3.0 | 45.9 ± 2.3 |
|  | future | 0.76 ± 0.02 | 7 ± 1 | 1.09 ± 0.03 | 185 ± 8 | 202 ± 6 | 6.2 ± 0.3 | 4.8 ± 0.1 | 29.9 ± 0.4 | 1.26 ± 0.03 | 49.0± 0.1 | 38.9 ± 0.8 |
|  | warming | 0.75 ± 0.03 | NA | 1.08 ± 0.04 | 203 ± 4 | 220 ± 5 | 7.4 ± 0.2 | 4.6 ± 0.4 | 27.5 ± 0.3 | NA | NA | NA |
| Strain X | present | 0.74 ± 0.03 | 17 ± 1 | 1.07 ± 0.04 | 187 ± 11 | 200 ± 9 | 7.5 ± 0.2 | 5.5 ± 0.3 | 25.0 ± 1.3 | 1.37 ± 0.10 | 38.8 ± 1.1 | 28.4 ± 2.7 |
|  | future | 0.71 ± 0.02 | 16 ± 2 | 1.02 ± 0.03 | 164 ± 5 | 167 ± 9 | 6.5 ± 0.4 | 4.2 ± 1.0 | 25.5 ± 2.5 | 1.31 ± 0.03 | 52.1 ± 2.0 | 39.9 ± 2.4 |
|  | warming | 0.8 ± 0.02 | NA | 1.15 ± 0.03 | 170 ± 16 | 196 ± 23 | 7.0 ± 1.0 | 4.4 ± 1.4 | 24.7 ± 4.0 | NA | NA | NA |
| Strain Y | present | 0.79 ± 0.01 | 10 ± 3 | 1.14 ± 0.01 | 281 ± 8 | 321 ± 12 | 11.2 ± 0.5 | 4.7 ± 0.1 | 25.1 ± 0.8 | 1.25 ± 0.02 | 46.8 ± 6.7 | 37.3 ± 4.7 |
|  | future | 0.82 ± 0.01 | 45 ± 2 | 1.19 ± 0.02 | 239 ± 2 | 284 ± 2 | 8.8 ± 0.4 | 4.8 ± 0.1 | 27.3 ± 1.3 | 1.04 ± 0.03 | 55.4 ± 6.4 | 53.5 ± 6.7 |
|  | warming | 0.73 ± 0.05 | NA | 1.05 ± 0.07 | 225 ± 11 | 236 ± 28 | 9.3 ± 0.6 | 4.8 ± 0.1 | 24.1 ± 0.4 | NA | NA | NA |
| Strain Z | present | 0.73 ± 0.03 | 13 ± 1 | 1.06 ± 0.04 | 344 ± 35 | 365 ± 44 | 10.4 ± 0.2 | 4.7 ± 0.1 | 33.2 ± 3.5 | 1.24 ± 0.03 | 66.0 ± 1.9 | 53.2 ± 2.9 |
|  | future | 0.8 ± 0.002 | 5 ± 0 | 1.15 ± 0.00 | 330 ± 37 | 380 ± 43 | 10.1 ± 0.1 | 4.8 ± 0.3 | 32.6 ± 3.2 | 1.28 ± 0.01 | 56.4 ± 1.4 | 44.1 ± 1.1 |
|  | warming | 0.71 ± 0.03 | NA | 1.02 ± 0.04 | 271 ± 6 | 276 ± 9 | 9.5 ± 0.5 | 5.2 ± 0.9 | 28.6 ± 0.9 | NA | NA | NA |
| Mean Monocultures | present | 0.77 ± 0.03 | NA | 1.11 ± 0.05 | 266 ± 55 | 296 ± 58 | 9.0 ± 2.0 | 5.0 ± 0.4 | 30 ± 5.1 | 1.32 ± 0.07 | 55.3 ± 10.3 | 42.2 ± 8.5 |
|  | future | 0.75 ± 0.03 | NA | 1.08 ± 0.05 | 219 ± 38 | 235 ± 36 | 7.9 ± 1.7 | 4.7 ± 0.3 | 29.4 ± 1.1 | 1.25 ± 0.11 | 54.9 ± 9.5 | 44.5 ± 8.9 |
|  | warming | 0.77 ± 0.01 | NA | 1.11 ± 0.01 | 238 ± 14 | 266 ± 15 | 8.2 ± 0.4 | 4.9 ± 0.4 | 28.0 ± 4.2 | NA | NA | NA |
| Multi-Strain | present | 0.73 ± 0.02 | NA | 1.06 ± 0.02 | 217 ± 5 | 230 ± 1 | 7.1 ± 0.2 | 4.9 ± 0.1 | 30.6 ± 0.7 | 1.29 ± 0.04 | 61.1 ± 3.9 | 47.6 ± 3.0 |
|  | future | 0.81 ± 0.02 | NA | 1.18 ± 0.02 | 187 ± 4 | 219 ± 5 | 6.8 ± 0.3 | 4.9 ± 0.1 | 27.5 ± 1.4 | 1.31 ± 0.03 | 60.9 ± 3.7 | 46.6 ± 2.4 |

**Table S2:** Carbonate chemistry of each culture as mean and standard deviation of biological replicates: pCO_2_ was calculated from measured pH and total alkalinity (TA) at the final time-point of incubation using CO_2_SYS (Pierrot et al., 2006) with the respective temperature, salinity of 32 and concentrations of 6.5 µmol kg^-1^ for phosphate and 100 µmol kg^-1^ for silicate.

| **Culture** | **Treatment** | **calculated pCO_2_**  **[µatm]** | **pH _NBS_** | **TA**  **[µmol kg^-1^]** |
| --- | --- | --- | --- | --- |
| **Strain A** | present | 378 ± 11 | 8.14 ± 0.01 | 2212 ± 18 |
| **Strain A** | high temp | 389 ± 12 | 8.13 ± 0.01 | 2123 ± 9 |
| **Strain A** | future | 1191 ± 25 | 7.70 ± 0.01 | 2232 ± 16 |
| **Strain B** | present | 377 ± 10 | 8.15 ± 0.01 | 2226 ± 7 |
| **Strain B** | high temp | 407 ± 9 | 8.11 ± 0.01 | 2114 ± 6 |
| **Strain B** | future | 1249 ± 18 | 7.68 ± 0.01 | 2205 ± 2 |
| **Strain C** | present | 339 ± 9 | 8.19 ± 0.01 | 2242 ± 10 |
| **Strain C** | high temp | 433 ± 5 | 8.09 ± 0.00 | 2123 ± 7 |
| **Strain C** | future | 1229 ± 30 | 7.69 ± 0.01 | 2237 ± 4 |
| **Strain X** | present | 414 ± 4 | 8.08 ± 0.00 | 2102 ± 2 |
| **Strain X** | high temp | 427 ± 4 | 8.09 ± 0.00 | 2114 ± 8 |
| **Strain X** | future | 1180 ± 33 | 7.70 ± 0.01 | 2237 ± 10 |
| **Strain Y** | present | 408 ± 6 | 8.09 ± 0.01 | 2121 ± 10 |
| **Strain Y** | high temp | 416 ± 11 | 8.1 ± 0.01 | 2116 ± 1 |
| **Strain Y** | future | 1152 ± 22 | 7.72 ± 0.01 | 2254 ± 13 |
| **Strain Z** | present | 396 ± 9 | 8.13 ± 0.01 | 2238 ± 2 |
| **Strain Z** | high temp | 412 ± 6 | 8.11 ± 0.01 | 2121 ± 9 |
| **Strain Z** | future | 1191 ± 11 | 7.70 ± 0.00 | 2222 ± 4 |
| **Multi-Strain** | present | 350 ± 7 | 8.18 ± 0.01 | 2263 ± 40 |
| **Multi-Strain** | future | 1095 ± 65 | 7.74 ± 0.03 | 2238 ± 16 |

**Table S3a:** Two-way-ANOVAs of single-strain monocultures testing the effect of strain identity, treatment and the interaction of both (strain*treatment) on cellular traits in all three treatments (present-day, high-temperature, future).µ describes exponential growth rate POC and Chl *a* are cell quota; alpha and ETR_max_ describe maximum light-use efficiency and maximum as absolute electron transport rate through photosystem II, respectively.

| **Test** | **dependent variable** | **Independent variable** | **F** | **p** | **sign (α=0.05)** |
| --- | --- | --- | --- | --- | --- |
| 2-way ANOVA | µ | strain | 3.7 | 0.009 | * |
|  |  | treatment | 6.2 | 0.005 | * |
|  |  | strain*treatment | 8.9 | 5.58e-07 | * |
| 2-way ANOVA | log(POC prod) | strain | 46.3 | 3.24e-14 | * |
|  |  | treatment | 31.9 | 1.57e-08 | * |
|  |  | strain*treatment | 6.3 | 2.11e-05 | * |
| 2-way ANOVA | Chl *a* | strain | 107.4 | <2e-16 | * |
|  |  | treatment | 22.8 | 5.34e-07 | * |
|  |  | strain*treatment | 7.7 | 2.94e-06 | * |
| 2-way ANOVA | log(POC) | strain | 92.3 | <2e-16 | * |
|  |  | treatment | 39.7 | 1.27e-09 | * |
|  |  | strain*treatment | 5.6 | 6.28e-05 | * |
| 2-way ANOVA | log(Chl *a*:POC) | strain | 31.1 | 9.22e-12 | * |
|  |  | treatment | 3.9 | 0.030 | * |
|  |  | strain*treatment | 5.8 | 4.55e-05 | * |
| 2-way ANOVA | log(alpha) | strain | 25.1 | 1.57e-07 | * |
|  |  | treatment | 23.4 | 1.32 e-04 | * |
|  |  | strain*treatment | 8.6 | 2.61 e-04 | * |
| 2-way ANOVA | log(ETR_max_) | strain | 21.4 | 5.34e-07 | * |
|  |  | treatment | 0.5 | 0.51 |  |
|  |  | strain*treatment | 14.5 | 8.95e-06 | * |

**Table S3b:** One-way-ANOVAs of multi-strain cultures comparing bulk responses measured in present and future scenario.

| **Test** | **dependent variable** | **F** | **p** | **sign (α=0.05)** |
| --- | --- | --- | --- | --- |
| One-way-ANOVA | µ | 62.7 | 0.000215 | * |
| One-way-ANOVA | POC prod | 3.99 | 0.0924 |  |
| One-way-ANOVA | POC | 84.0 | 9.50E-05 | * |
| One-way-ANOVA | alpha | 0.80 | 0.406 |  |
| One-way-ANOVA | In-situ ETR | 0.5 | 0.491 |  |
| One-way-ANOVA | ETR_max_ | 0.002 | 0.966 |  |
| One-way-ANOVA | evenness | 100.9 | 0.000167 | * |

**Table S3c:** One-way-ANOVAs of multi-strain cultures comparing measured bulk responses with predictions based on strain composition and monoculture measurements as depicted in Table 2.

| **Test** | **dependent variable** | **F** | **p** | **sign (α=0.05)** |
| --- | --- | --- | --- | --- |
| One-way-ANOVA | Present-day µ | 2.8 | 0.147 |  |
| One-way-ANOVA | Present-day Chl *a* | 83.8 | 9.58E-05 | * |
| One-way-ANOVA | Present-day POC | 57.4 | 2.75E-04 | * |
| One-way-ANOVA | Present-day C:N (mol) | 0.3 | 0.616 |  |
| One-way-ANOVA | Present-day Chl *a*:POC | 9.1 | 0.024 | * |
| One-way-ANOVA | Present-day POC prod | 39.2 | 7.70E-04 | * |
| One-way-ANOVA | Future µ | 30.3 | 0.002 | * |
| One-way-ANOVA | Future Chl *a* | 52.1 | 3.58E-04 | * |
| One-way-ANOVA | Future POC | 332.4 | 1.75E-06 | * |
| One-way-ANOVA | Future C:N (mol) | 6.0 | 0.050 | * |
| One-way-ANOVA | Future Chl *a*:POC | 3.3 | 0.121 |  |
| One-way-ANOVA | Future POC prod | 139.3 | 2.24E-05 | * |

**Table S4:** Standardized effect size in mono- and multi-strain-culture growth rates caused by treatment (warming and future vs. present-day), by strain identity (strain growth vs. strain mean growth of all monocultures) and by diversity (observed growth in multi-strain culture vs. in monoculture). Standardized effect size was calculated following Borenstein et al. 2009 (raw mean difference of effect/ pooled standard deviation).

| **Strain** | **Standardized effect size** | | | | | | |
| --- | --- | --- | --- | --- | --- | --- | --- |
|  | **Treatment** | | **Strain mean** | | | **Diversity** | |
|  | **warming** | **future** | **present-day** | **warming** | **future** | **present-day** | **future** |
| Strain A | 0.42 | -2.62 | -0.68 | 3.39 | -5.71 | -2.56 | 2.11 |
| Strain B | -2.44 | 0.47 | 0.44 | -0.87 | 1.11 | -2.14 | -0.59 |
| Strain C | -2.30 | -2.46 | 1.61 | 0.07 | -0.63 | -3.00 | 0.61 |
| Strain X | 2.51 | -1.51 | -1.30 | 2.22 | -3.53 | -1.43 | 7.04 |
| Strain Y | -1.56 | 3.62 | 2.81 | -0.41 | 5.04 | -4.99 | 2.43 |
| Strain Z | -1.08 | 3.44 | -1.44 | -1.51 | 11.91 | -0.15 | -6.65 |
| Multi-strain | NA | 5.25 | -2.55 | NA | 2.91 | NA | NA |

**References**

Excoffier, L., Lischer, H. E. L. (2010). Arlequin suite ver 3.5: a new series of programs to perform population genetics analyses under Linux and Windows. *Molecular Ecology Resources,* 10 (3), 564-567. https://doi.org/10.1111/j.1755-0998.2010.02847.x

John, U., Tillmann, U., Hülskötter, J., Alpermann, T. J., Wohlrab, S., Van De Waal, D. B. (2015). Intraspecific facilitation by allelochemical mediated grazing protection within a toxigenic dinoflagellate population. *Proceedings of the Royal Society of London B: Biological Sciences,* 282 (1798), 20141268. https://doi.org/10.1098/rspb.2014.1268

Li, H., Durbin, R. (2009). Fast and accurate short read alignment with Burrows–Wheeler transform. *Bioinformatics,* 25 (14), 1754-1760. 10.1093/bioinformatics/btp324

Li, H., Handsaker, B., Wysoker, A. *et al.* (2009). The Sequence Alignment/Map format and SAMtools. *Bioinformatics,* 25 (16), 2078-2079. https://doi.org/10.1093/bioinformatics/btp352

Mayer, C., Leese, F., Tollrian, R. (2010). Genome-wide analysis of tandem repeats in *Daphnia pulex* - a comparative approach. *BMC Genomics,* 11 (1), 277. https://doi.org/10.1186/1471-2164-11-277

Meyer, J. R., Ellner, S. P., Hairston, N. G., Jones, L. E., Yoshida, T. (2006). Prey evolution on the time scale of predator–prey dynamics revealed by allele-specific quantitative PCR. *Proceedings of the National Academy of Sciences,* 103 (28), 10690-10695. https://doi.org/10.1073/pnas.0600434103

Pierrot, D. E., Lewis, E., Wallace, D. W. R. (2006) MS Exel Program Developed for CO_2_ System Calculations. (ed Ornl/Cdiac-105acarbon Dioxide Information Analysis Centre, O. R. N. L.), US Department of Energy.

Wolf, K. K. E., Hoppe, C. J., Leese, F., Rost, B., John, U. (in prep.).
